# Addition of Exogenous NAD^+^ Prevents Mefloquine-Induced Neuroaxonal and Hair Cell Degeneration through Reduction of Caspase-3-Mediated Apoptosis in Cochlear Organotypic Cultures

**DOI:** 10.1371/journal.pone.0079817

**Published:** 2013-11-06

**Authors:** Dalian Ding, Weidong Qi, Dongzhen Yu, Haiyan Jiang, Chul Han, Mi-Jung Kim, Kana Katsuno, Yun Hua Hsieh, Takuya Miyakawa, Richard Salvi, Masaru Tanokura, Shinichi Someya

**Affiliations:** 1 Center for Hearing and Deafness, State University of New York at Buffalo, Buffalo, New York, United States of America; 2 Sixth People’s Hospital, Shanghai Oriental Otolaryngology Institute, Shanghai Jiao Tong University, Shanghai, China; 3 Xiangya Hospital, Central South University, Changsha, Hunan, China; 4 Department of Applied Biological Chemistry, University of Tokyo, Yayoi, Tokyo, Japan; 5 Department of Otolaryngology-Head and Neck Surgery, Huashan Hospital, Fudan University, Shanghai, China; 6 Departments of Aging and Geriatric Research, Division of Biology of Aging, University of Florida, Gainesville, Florida, United States of America; Pomona College, United States of America

## Abstract

**Background:**

Mefloquine is widely used for the treatment of malaria. However, this drug is known to induce neurological side effects including depression, anxiety, balance disorder, and sensorineural hearing loss. Yet, there is currently no treatment for these side effects.

**Principal Findings:**

In this study, we show that the coenzyme NAD^+^, known to play a critical role in maintaining the appropriate cellular redox environment, protects cochlear axons and sensory hair cells from mefloquine-induced degeneration in cultured rat cochleae. Mefloquine alone destroyed hair cells and nerve fiber axons in rat cochlear organotypics cultures in a dose-dependent manner, while treatment with NAD^+^ protected axons and hair cells from mefloquine-induced degeneration. Furthermore, cochlear organs treated with mefloquine showed increased oxidative stress marker levels, including superoxide and protein carbonyl, and increased apoptosis marker levels, including TUNEL-positive nuclei and caspases-3. Treatment with NAD^+^ reduced the levels of these oxidative stress and apoptosis markers.

**Conclusions/Significance:**

Taken together, our findings suggest that that mefloquine disrupts the cellular redox environment and induces oxidative stress in cochlear hair cells and nerve fibers leading to caspases-3-mediated apoptosis of these structures. Exogenous NAD^+^ suppresses mefloquine-induced oxidative stress and prevents the degeneration of cochlear axons and sensory hair cells caused by mefloquine treatment.

## Introduction

Axonal degeneration is a common pathological feature of a variety of neuropathological disorders, such as Alzheimer’s disease, amyotrophic lateral sclerosis, Parkinson’s disease, and diabetic neuropathies [1-3], and is recognized as a potentially important therapeutic target. Nevertheless, the development of effective therapeutic interventions has been hampered by a detailed understanding of the mechanisms by which axon degeneration occurs with various neurodegenerative diseases. Mefloquine (Lariam ®) is an antimalarial drug that is widely used for prophylactic and therapeutic treatment of malaria [4-6]; however, it can induce a range of neurological side effects such as anxiety, panic attacks, nightmares, dizziness, tremor, headache, fatigue, grand mal seizures, suicidal ideation, balance disturbance, and hearing loss [7-9]. Several *in vitro* studies have revealed that mefloquine selectively damaged cochlear and vestibular hair cells and spiral ganglion neurons through apoptosis [10-13]. Moreover, mefloquine is also known to cause primary rat cortical neuron degeneration, neuronal calcium homeostasis disruption in embryonic rat neurons, and significant axonal degeneration [14-16]. Taken together, these results exemplify its ototoxic and neurotoxic potential. 

Evidence indicates that the depletion of the coenzyme nicotinamide adenine dinucleotide (NAD^+^) results in axonal degeneration [17,18]. Moreover, NAD^+^ supplementation suppresses the development of axonal degeneration in traumatic injury, ischemia damage, autoimmune encephalomyelitis, p53-induced neuron apoptosis, and radiation-induced immunosuppression [19-21]. The reduction of axonal degeneration by NAD^+^ is presumably due to its propensity to reduce oxidative stress or oxidative damage in the neurons [22-24]. 

To gain a better understanding of the mechanisms involved in mefloquine ototoxic damage and potential therapeutic interventions, we treated cochlear organotypic cultures with various doses of mefloquine and examined the samples for evidence of oxidative stress and cellular pathways involved in programmed cell death. We show that mefloquine destroys cochlear hair cells and axons in a dose-dependent manner. Cochlear cultures treated with mefloquine display increased levels of oxidative stress markers, including supuroxide, one of the most well studied ROS (reactive oxygen species), and protein carbonyl, a protein oxidative marker. Mefloquine treatment also increased levels of apoptosis markers, including TUNEL-positive nuclei, a nuclear DNA fragmentation marker, and caspases-3, one of the most well studied members of the cysteine protease family. Interestingly, treatment with NAD^+^ reduced the levels of oxidative stress and apoptosis markers and prevented mefloquine-induced degeneration of cochlear axons and sensory hair cells.

## Materials and Methods

### Animal Procedures

Sprague-Dawley rat pups were purchased from Charles River Laboratories (Wilmington, MA). All experimental procedures were approved by the Institutional Animal Care and Use Committee (IACUC) of University at Buffalo. 

### Reagents

Mefloquine hydrochloride (M2319), β- nicotinamide adenine dinucleotide hydrate (N1511), mouse monoclonal antibody against neurofilament 200 (N0142), TRITC conjugated goat anti-mouse IgG secondary antibody (T5393), and dihydroethidium (D7008) were purchased from Sigma-Aldrich (St. Louise, MO). Alexa Fluor® 488 phalloidin (A12379) and TO-PRO-3 (T3605) were purchased from Invitrogen (Grand Island, NY). OxyBlot^tm^ protein oxidation detection kit (S7150) was purchased from Millipore (Billerica, MA). APO-BrdU TUNEL assay kit was purchased from Molecular Probes (Eugene, OR). CaspGLOW Red active caspase-3 staining kit was purchased from BioVision (Milpitas, California). All other reagents were purchased from Sigma unless otherwise indicated.

### Cochlear organotypic cultures

The procedures for preparing cochlear organotypic cultures have been previously described [25-27]. Briefly, on postnatal day 3, rat pups were decapitated. The cochleae were then removed and placed in Hank’s balanced salt solution (1X GIBCO, 14175, Invitrogen, Carlsbad, CA). After removing the lateral wall, the whole basilar membrane containing the organ of Corti and spiral ganglion neurons was isolated, transferred onto a collagen gel matrix in a 35 mm culture dish containing a solution consisting of 15 μl of rat tail collagen (Type 1, BD Biosciences, 4236 Bedford, MA, 10×basal medium eagle (BME), Sigma B9638, 2% sodium carbonate, 9:1:1 ratio) and 1.3 ml of serum-free medium consisting of 2 g bovine serum albumin (BSA, Sigma A-4919), 2 ml serum-free supplement (Sigma I-1884), 4.8 ml of 20% glucose (Sigma G-2020), 0.4 ml penicillin G (Sigma P-3414), 2 ml of 200 mM glutamine (Sigma G-6392), and 190.8 ml of 1X BME (Sigma B-1522), and incubated overnight (Forma Scientific 3029, 37°C in 5% CO_2_). 

### Mefloquine and NAD^+^ treatment

Cultured cochleae were treated with 35 µM or 50 µM mefloquine for 24 hours to induce degeneration of auditory nerve fibers and cochlear hair cells. To investigate if NAD^+^ protected the auditory neurons and cochlear hair cells from mefloquine-induced degeneration, cultured cochleae were treated with 5mM or 20mM NAD^+^, while controls were cultured without NAD^+^ in standard serum-free medium. 

### Histology

Twenty four hours after mefloquine or NAD^+^ treatments, the cochlear explants were fixed with 10% phosphate buffered formalin for 1 hour. The specimens were then rinsed in 0.01 M PBS and incubated overnight (4°C) in a solution consisting of 20 µl of mouse anti-neurofilament 200 kD antibody (Sigma N0142, clone N52), 20 µl Triton X-100 (10%), 6 µl normal goat serum, 154 µl of 0.01 M PBS. Next, the specimens were rinsed with 0.01 M PBS, immersed in a solution consisting of 2 µl of secondary anti-mouse IgG TRITC (Sigma T5393), 12 µl normal goat serum, 40 µl Triton X-100 (10%) and 345 µl of 0.01 M PBS, rinsed with 0.01 M PBS, and double-stained with Alexa Fluor 488 phalloidin (Invitrogen A12379) for 30 minutes. The specimens were then rinsed in 0.01M PBS, mounted on glass slides in glycerin, coverslipped, and examined with a confocal microscope (Zeiss LSM-510) with appropriate filters for TRITC (absorption: 544 nm, emission: 572 nm) to visualize the auditory nerve fibers and spiral ganglion neurons and for Alexa Fluor 488 (excitation 495 nm, emission 519 nm) to visualize the stereocilia and cuticular plate of the hair cells. Images were evaluated with a Zeiss LSM Image Examiner; images were post-processed with Adobe Photoshop software.

### Quantification of auditory nerve fibers

The numbers of auditory nerve fibers projecting to the cochlear hair cells were counted in the basal and apical turns using a fluorescence microscope at 600x magnification as previously described [28]. Nerve fiber counts were made on 6cochlear explants per experimental condition (*n* = 6). 

### Cytocochleograms

The number of inner hair cells (IHC), first-row outer hair cells (OHC1), second-row outer hair cells (OHC2), and third-row outer hair cells (OHC3) were counted over 0.24 mm intervals along the entire length of the cochlea using a microscope at 400x magnification. The countswere entered into a custom computer program designed to compute a cochleogram showing the number of missing IHC and OHC (average loss in rows1-3) as a function of percentage distance from the apex of the cochlea [29-31]. 

### Quantification of cochlear hair cells

The cochleogram program was also used to compute the percentage missing hair cells in 10% intervals along the length of the cochlea starting from the apex. For each cochlea, the mean density of OHCs and IHCs was determined in the apical region (10%-20% distance from the cochlear apex) or in the basal region (70%-80% distance from the cochlear apex). 

### Superoxide detection

Dihydroethidium (Sigma D7008) was used to label superoxide in cultured cochlear organs. To measure superoxide levels, cultured cells were treated with 100 nM dihydroethidium for 30 minutes. After 2 hours fixation with 10% formalin in PBS, the hair cells were stained with Alexa Fluor® 488 phalloidin for 30 minutes, while nuclei were stained with 0.75 µM To-PRO-3 for 20 minutes. The stained sections were examined with a confocal microscope (Zeiss LSM-510) using appropriate filters to detect dihydroethidium/superoxide (absorption: 544 nm, emission: 572 nm), Alexa-Fluor 488/phalloidin which is heavily expressed in the stereocilia of cochlear hair cells (excitation 495 nm, emission 519 nm), and TO-PRO-3 labeled nuclei (excitation 642 nm, emission 661 nm). Images were evaluated with the Zeiss LSM Image Examiner and Adobe Photoshop software. Red fluorescent signals were quantified using ImageJ software (ver. 1.46j) as follows. The image files were split to three images (Green, Red and Blue) using Image/split channels. The red image, representing superoxide labeling, was chosen and from the analyze menu, the function “measure” was used to obtain values of integrated intensity in the designated area. In order to get more exact values of integrated intensity, mean values of background intensity were subtracted. Mean values of integrated intensity/area were determined for each group (*n*=3) and used to compute the average superoxide level. 

### Protein oxidation

To investigate protein oxidation levels, protein carbonyl levels were measured using the OxyBlot oxidized protein detection kit (Millipore) according to the manufacturers instructions. Briefly, after mefloquine treatment for 12 hours, two cochlear basilar membranes, including sensory hair cells, supporting cells, and spiral ganglion neurons were lysed with 150 µl RIPA lysis buffer (Sigma-Aldrich) on ice for 30 minutes. Samples were run in triplicate and the intensity of the bands was quantified using the ImageJ (1.46J) software. 

### Quantification of NAD^+^ biosynthetic enzymes expression by quantitative RT-PCR

To measure mRNA expression levels of NAD^+^ biosynthetic enzymes, cochlear explants were treated with 50 µM mefloquine alone or without mefloquine as controls for 6 hours. Cochlear tissues were then homogenized for 30 seconds using a BioMasher II (Nippi) in SV RNA lysis buffer (Promega). Total RNA was then extracted and purified using the SV Total RNA Isolation System (Promega) according to the manufacturer’s instructions. The concentration and purity of isolated RNA was quantified using the NanoDrop™ 1000 spectrophotometer (Thermo Scientific). cDNA was synthesized from 1 μg of total RNA in a 20 μl solution with an oligo d(T)_18_ primer using the iScript cDNA Synthesis kit (Bio-Rad) according to the manufacturer’s instructions, and stored at −80 °C until required.

Quantitative real-time PCR (qRT-PCR) was performed in duplicate using the SYBR Premix EX Taq II (Takara) on the CFX96^TM^ real-time PCR detection system (Bio-Rad). Each 25 µL reaction solution contained 2.5 µl of 20-fold-diluted cDNA solution and primers at the final concentration of 200 nM. PCR conditions were as follows: 95°C for 30 seconds, then 39 cycles of 95°C for 5 seconds, and 60°C for 30 seconds. The primer sequences and their annealing temperatures are given in Table 1. Relative quantification analysis of the qRT-PCR data was performed using the CFX96^TM^ real-time PCR software (Bio-Rad) based on the comparative ΔΔCt method with *Gapdh* as the reference gene. Finally, a mean expression ratio of the target genes in each treated cochlear tissue was normalized to that of the non-treated cochlear tissue. 

**Table 1:** Primer sequences and annealing temperature for qRT-PCR.

Gene	Forward primer	Reverse primer	Annealing temperature (°C)
*NAPRT*	AGGACTGTATGCGCTTTCTTC	CCAGAGCAATCAAGGGCTCG	60
*NMPRT*	GCAGAAGCCGAGTTCAACATC	TTTTCACGGCATTCAAAGTAGGA	60
*QNS*	CTCCTGCATTCGCTCCAAGTT	CCGGTGCATTATAGGCATCCC	60
*NmNAT1*	TTCAAGGCCTGACAACATCGC	GAGCACCTTCACAGTCTCCACC	53
*NmNAT2*	ATGACCGAGACCACAAAGACC	ACAATCCCGCCAATCACAATAA	53
*NmNAT3*	ATCACGAATATGCACCTGCG	ATTGACGGGTGAGATGATGCC	63
*Nrk1*	TCATTGGAATTGGTGGTGTGAC	CAACAGGAAACTGCTGACATCAT	63 (for 1 min)
*Nrk2*	CTCGGACACCCACGTACTC	CACGGTCAGGAAGTAGCGTT	62
*QPRT*	CTGGACAACCTCACCCAGTT	GTCCATTCTGGTGGCATCTT	63

### Measurement of NAD^+^ levels and NAD^+^/NADH ratios

NAD^+^ levels, NADH levels, and NAD^+^/NADH ratios were measured from cochlear rat cultures without mefloquine treatment (untreated) or treated with 35 µM mefloquine using the NAD^+^/NADH quantification kit (BioVision, Milpitas, CA) according to the manufacturer’s instructions.

### TUNEL

Fifteen cochlear explants were divided into three groups: normal control, 35 μM mefloquine, and 35 μM mefloquine plus 20 mM NAD^+^. The cultures were treated for 6 hours and then fixed with 10% formalin in PBS for 4 hour. The APO-BrdU TUNEL assay kit (Molecular Probes A-23210) was used to evaluate nuclear DNA fragmentation according to the manufacturer’s protocol. Briefly, the fixed cochlear explants were transferred to ethanol overnight (4 °C), rinsed in wash buffer from the kit, and then incubated with DNA-labeling solution overnight (4 °C). Afterwards, specimens were rinsed twice in Rinse buffer from the kit and then incubated in 100 µl of freshly prepared antibody labeling solution by mixing with 5 µl of the Alexa Fluor 488-labeled anti-BrdU antibody with 95 µl of Rinse buffer for 60 minutes. The specimens were routinely double labeled with mouse anti-neurofilament 200 kD antibody (Sigma N0142, clone N52) followed by secondary anti-mouse IgG TRITC (Sigma T5393). The nuclei were also stained with TO-PRO-3 as described above. Images were collected with a confocal microscope (Zeiss LSM-510) with appropriate filters for red fluorescence of spiral ganglion neurons by TRITC-neuronfilament labeling, green fluorescence of positive TUNEL labeling, and blue fluorescence of TO-PRO-3 nuclei labeling, and evaluated with software (Zeiss LSM Image Examiner, Adobe Photoshop). To quantify TUNEL-positive spiral ganglion neurons, the total number of spiral ganglion neurons and the number of TUNEL-positive neurons were counted in each image. Counts were obtained from 5 cochlear explants per condition. For each condition, counts of spiral ganglion neurons were obtained from three locations in the middle turn of each cochlear explant. In each location, spiral ganglion neurons were counted in a 141.4 X 141.4 µm square area (optical section thickness 1 µm); counts were obtained in 2 optical sections separated by 20 µm to prevent double counting of neurons. 

### Caspase-3

Our previous studies demonstrated that executioner caspase-3 plays an important role in mefloquine-induced apoptosis in cochlear hair cells and spiral ganglion neurons [32-35]. To evaluate caspase-3 activity in live cells, a novel cell membrane-permeable fluorogenic caspase substrate, Red-IETD-FMK caspase-3 (BioVision Lot 70693), was used to label spiral ganglion neurons treated with 35 µM mefloquine or treated with 35 µM mefloquine plus 20 mM NAD^+^. Six hours after treatment, spiral ganglion neurons were incubated with Red-IETD-FMK caspase-3 diluted 1:200 for 60 minutes, and then fixed in 10% formalin in PBS for 24 hours. After rinsing with PBS, specimens were labeled with anti-neurofilament 200 kD antibody as described above. Confocal microscopy was performed using appropriate filters to stimulate red fluorescence of active caspase-3 and green fluorescence of spiral ganglion neurons simultaneously, and images were evaluated with software (Zeiss LSM Image Examiner, Adobe Photoshop). The quantification of active caspase-3-positive cells was performed in the same manner as that of TUNEL-positive cells. Briefly, the total number of spiral ganglion neurons in collected images and the number of active caspase-3-positive neurons were counted. Counts were obtained from 3 locations in the middle turn of each cochlear explant for five conditions. The image area is 141.4 X 141.4 µm (optical section thickness 1 µm). The counts were obtained in 2 optical sections separated by 20 µm. 

### Statistical analysis

All Statistical analyses were carried out by one-way ANOVA, followed by Newman-Keuls post hoc analyses using the GraphPad Prism 5 software [31]. All tests were two-sided with the threshold for statistical significance set at *P* < 0.05.

## Results

### 1: Mefloquine causes degeneration of cochlear hair cells and auditory nerve fibers

**Figure 1:**
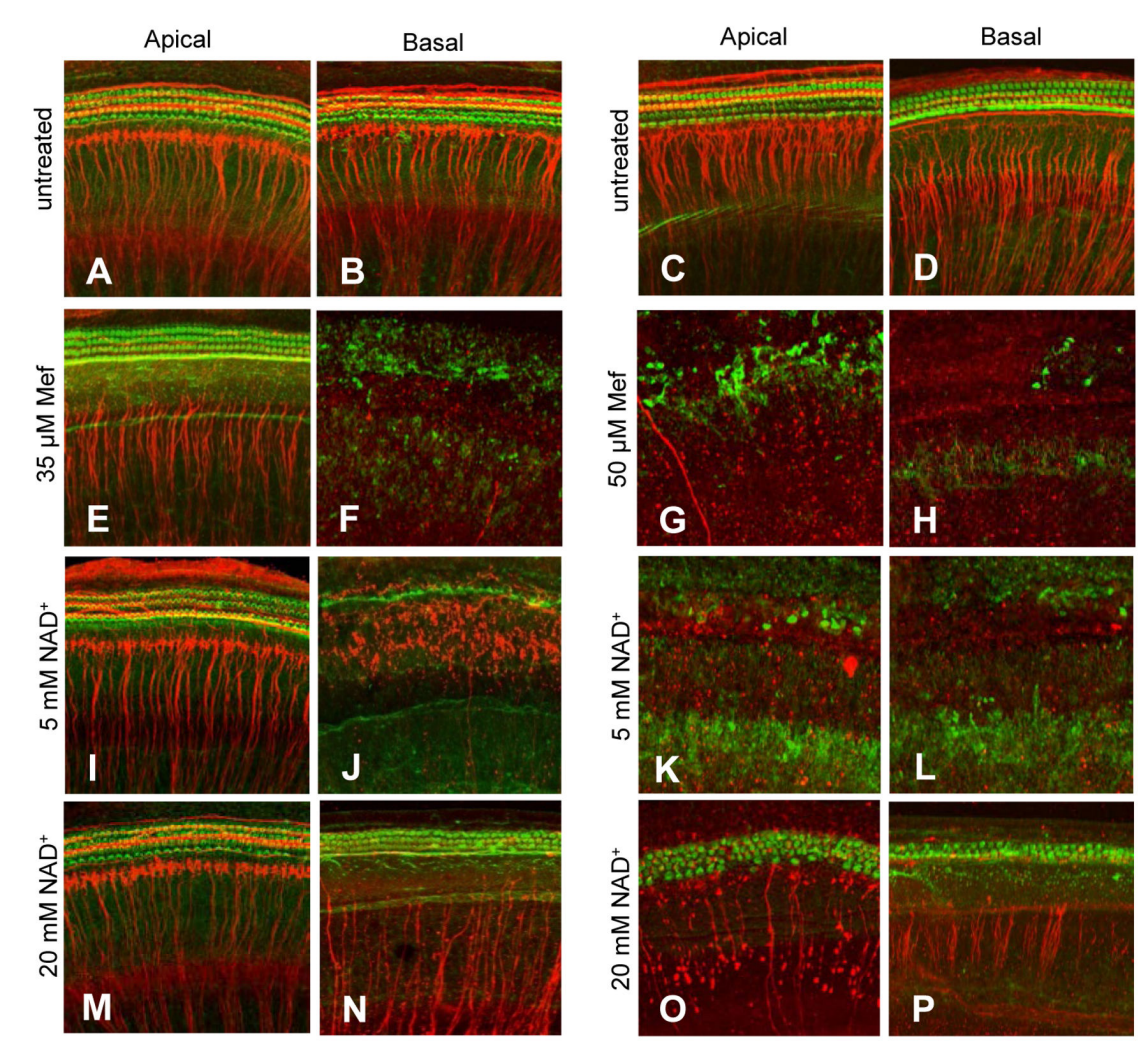
Mefloquine treatment causes degeneration of cochlear hair cells and auditory nerves, while NAD^+^ reduces the degeneration. Hair cells (green fluorescence) and auditory nerve fibers (red fluorescence) in the apical turn and the basal turn of the cultured cochlea without mefloquine treatment (untreated) (a-d), or treated with 35 µM mefloquine alone (e-f), 50 µM mefloquine alone (g-h), 35 µM mefloquine + 5 mM NAD^+^ (i-j), 50 µM mefloquine + 5 mM NAD^+^ (k-l), 35 µM mefloquine + 20 mM NAD^+^ (m-n) or 50 µM mefloquine + 20 mM NAD^+^ (o-p).

**Figure 2:**
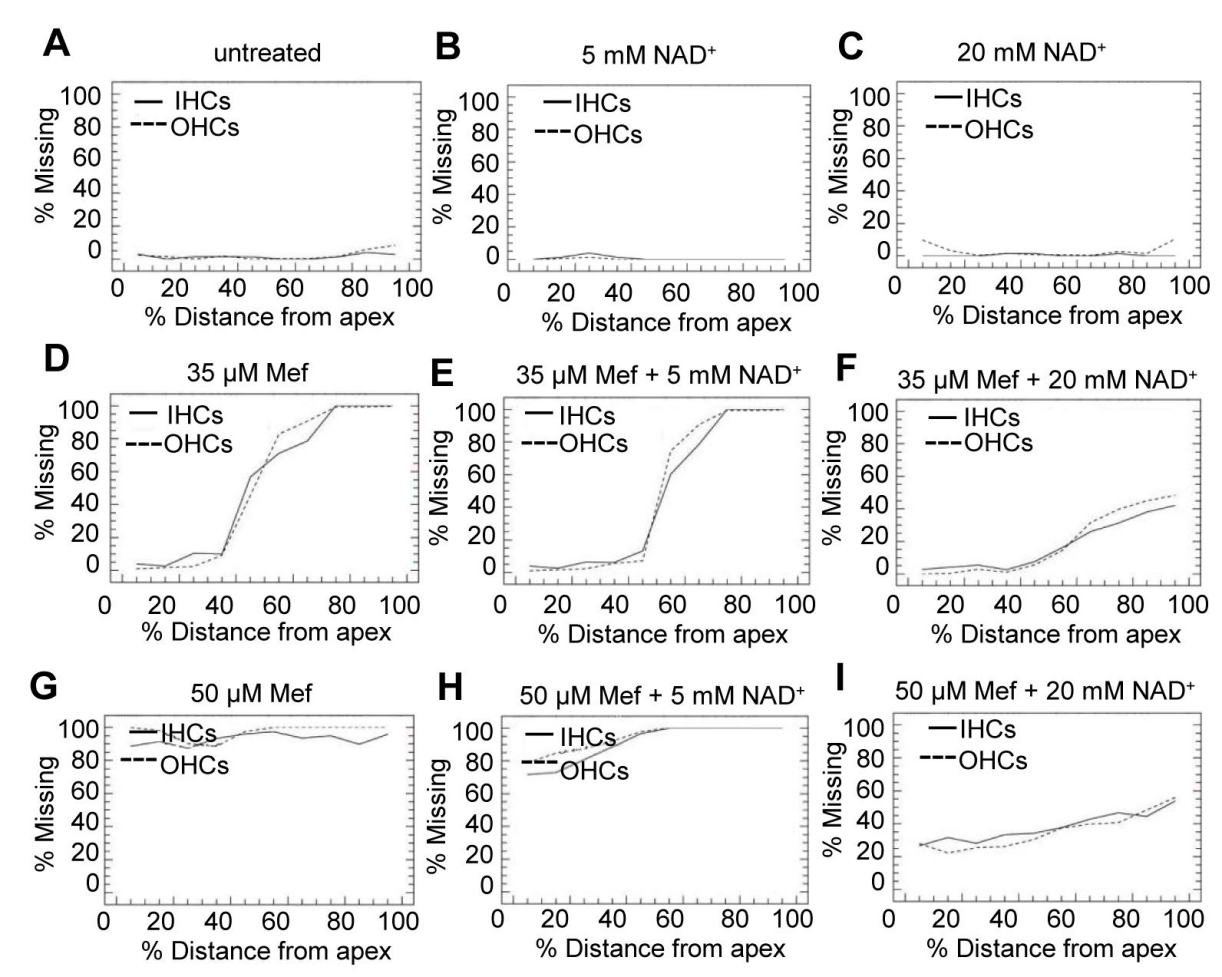
Mefloquine treatment decreases cochlear outer and inner hair cell survival, while NAD^+^ treatment protects the hair cells. Averaged cochleogram from untreated (a), 5 mM NAD^+^ treatment (b), 20 mM NAD^+^ treatment (c), 35 µM mefloquine treatment (d), 50 µM mefloquine treatment (e), 35 µM mefloquine + 5 mM NAD^+^ treatment (f), 35 µM mefloquine + 20 mM NAD^+^ (g), 50 µM mefloquine + 5 mM NAD^+^ (h) or 50 µM mefloquine + 20 mM NAD^+^ (i).

**Figure 3:**
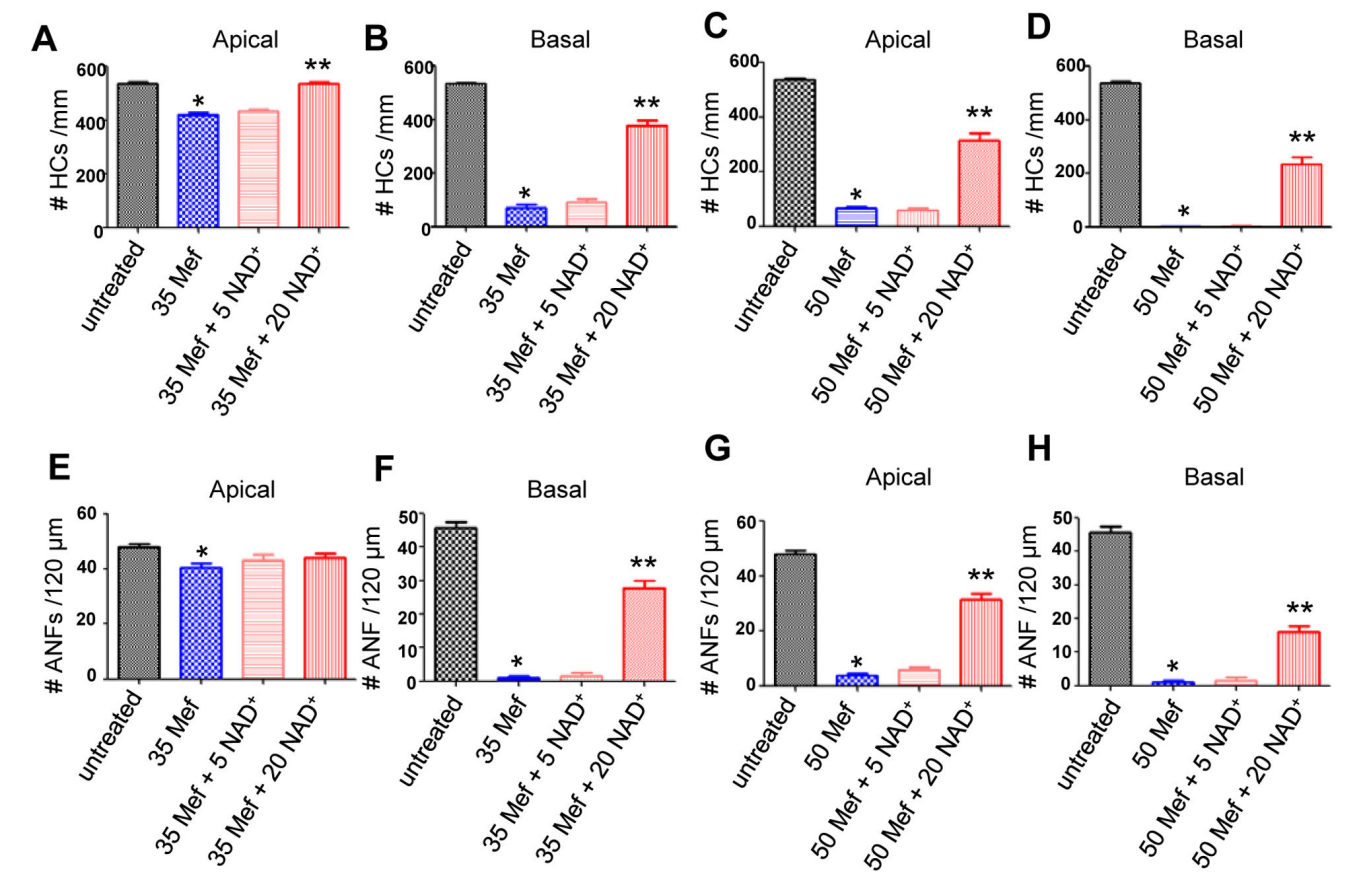
Mefloquine treatment reduces cochlear hair cell and auditory nerve fiber density, while NAD^+^ treatment prevents the decline. Hair cell (HC) density in the apical and basal turn of the cultured cochlea treated with 35 µM mefloquine alone, 35 µM mefloquine + 5 mM NAD^+^, or 35 µM mefloquine + 20 mM NAD^+^ (a-b) or 50 µM mefloquine alone, 50 µM mefloquine + 5 mM NAD^+^, or 50 µM mefloquine + 20 mM NAD^+^ (c-d). Auditory nerve fiber (ANF) density in the apical and basal turn of the cultured cochlea treated with 35 µM mefloquine alone, 35 µM mefloquine + 5 mM NAD^+^, or 35 µM mefloquine + 20 mM NAD^+^ (e-f) or 50 µM mefloquine alone, 50 µM mefloquine + 5 mM NAD^+^, or 50 µM mefloquine + 20 mM NAD+ (g-h). *Significantly different from control (P < 0.05). **Significantly different from mefloquine treatment alone (*P* < 0.05). 35 Mef = 35 µM mefloquine, 50 Mef = 50 µM mefloquine, 5 NAD^+^ = 5 mM NAD^+^, and 20 NAD^+^ = 20 mM NAD^+^.

To investigate whether mefloquine induces degeneration of the hair cells and auditory nerve fibers in a dose-dependent manner, cultured rat cochleae were treated with 0, 35 µM, or 50 µM mefloquine, and then double-stained with Alexa Fluor 488 phalloidin to visualize the stereocilia bundles and cuticular plate of the hair cells, and mouse anti-neurofilament 200 kD antibody followed by anti-mouse IgG conjugated to visualize the auditory nerve fibers. We first confirmed that untreated controls (no mefloquine treatment) displayed no obvious degeneration of the cochlear hair cells (green) and auditory nerve fibers (red) in the apical (Figure 1a and c) and basal regions (Figure 1b and d). Treatment with 35 µM mefloquine for 24 hours resulted in significant loss of cochlear hair cells and auditory nerve fibers in the basal turn (Figure 1f), and loss of the auditory nerve fibers that synapse beneath the IHCs in the apical turn (Figure 1e). Treatment with 50 µM mefloquine for 24 hours completely destroyed most of the hair cells and auditory nerve fibers in both the apex and base of the cochlea (Figure 1g-h). Next, we quantified the numbers of hair cells and nerve fibers in the apical and basal regions in cultured rat cochleae. We found that treatment with 35 µM mefloquine moderately decreased the numbers of hair cells in the apical region (Figure 2d and Figure 3a-b), while 50 µM mefloquine severely decreased the numbers of hair cells in both the apical and basal regions (Figure 2g and Figure 3c-d). Moreover, 50 µM mefloquine severely decreased the numbers of auditory nerve fibers in both the apical and basal regions (Figure 3g-h). Together, these results show that mefloquine induces degeneration of auditory nerve fibers and hair cells in cultured cochleae in a dose-dependent manner.

### 2: NAD^+^ protects cochlear hair cells and auditory nerve fibers from mefloquine-induced degeneration

Yang et al. have shown that an increase in NAD^+^ levels promotes cell survival under cytotoxic stress [36]. Hence, we hypothesized that treatment with NAD^+^ may protect sensory hair cells and auditory nerve fibers from mefloquine-induced degeneration. To test this hypothesis, cultured rat cochleae were treated with mefloquine alone or both mefloquine and NAD^+^ at various concentrations. Addition of 5 mM NAD^+^ to cochlear cultures treated with 35 µM mefloquine protected the nerve fibers that synapse beneath the IHCs in the apical turn (Figure 1i), but did not protect the hair cells and auditory nerve fibers in the basal turn (Figure 1j). Under 50 µM mefloquine treatment, addition of 5 mM NAD^+^ had no protective effects on the cochlear hair cells and nerve fibers in both the apical turn and basal turn (Figure 1k-l). However, addition of 20 mM NAD^+^ to cochlear cultured treated with either 35 µM or 50 µM mefloquine protected the auditory nerve fibers and cochlear hair cells in both the apical turn and basal turn (Figure 1m-p).We then quantified the numbers of hair cells and nerve fibers in the apical and basal regions in cultured rat cochleae treated with mefloquine alone, NAD^+^ alone, or treated with both mefloquine and NAD^+^. We first confirmed that treatment with 5 mM or 20 mM NAD^+^ did not affect hair cell numbers (Figure 2b-c). We then found that treatment with 20 mM NAD^+^, but not 5 mM NAD^+^, significantly increased hair cell numbers under either 35 µM or 50 µM mefloquine-treatment (Figure 2e-f, h-I and Figure 3a-d). Treatment with 20 mM NAD^+^, but not 5 mM NAD^+^, also increased auditory nerve fiber survival under either 35 µM or 50 µM mefloquine-treatment (Figure 3f-h). Together, these results indicate that NAD^+^ treatment protects sensory hair cells and auditory nerve fibers from mefloquine-induced degeneration in a dose-dependant manner. 

### 3: NAD^+^ reduces mefloquine-induced oxidative stress in the auditory nerves and hair cells in cultured cochleae

**Figure 4:**
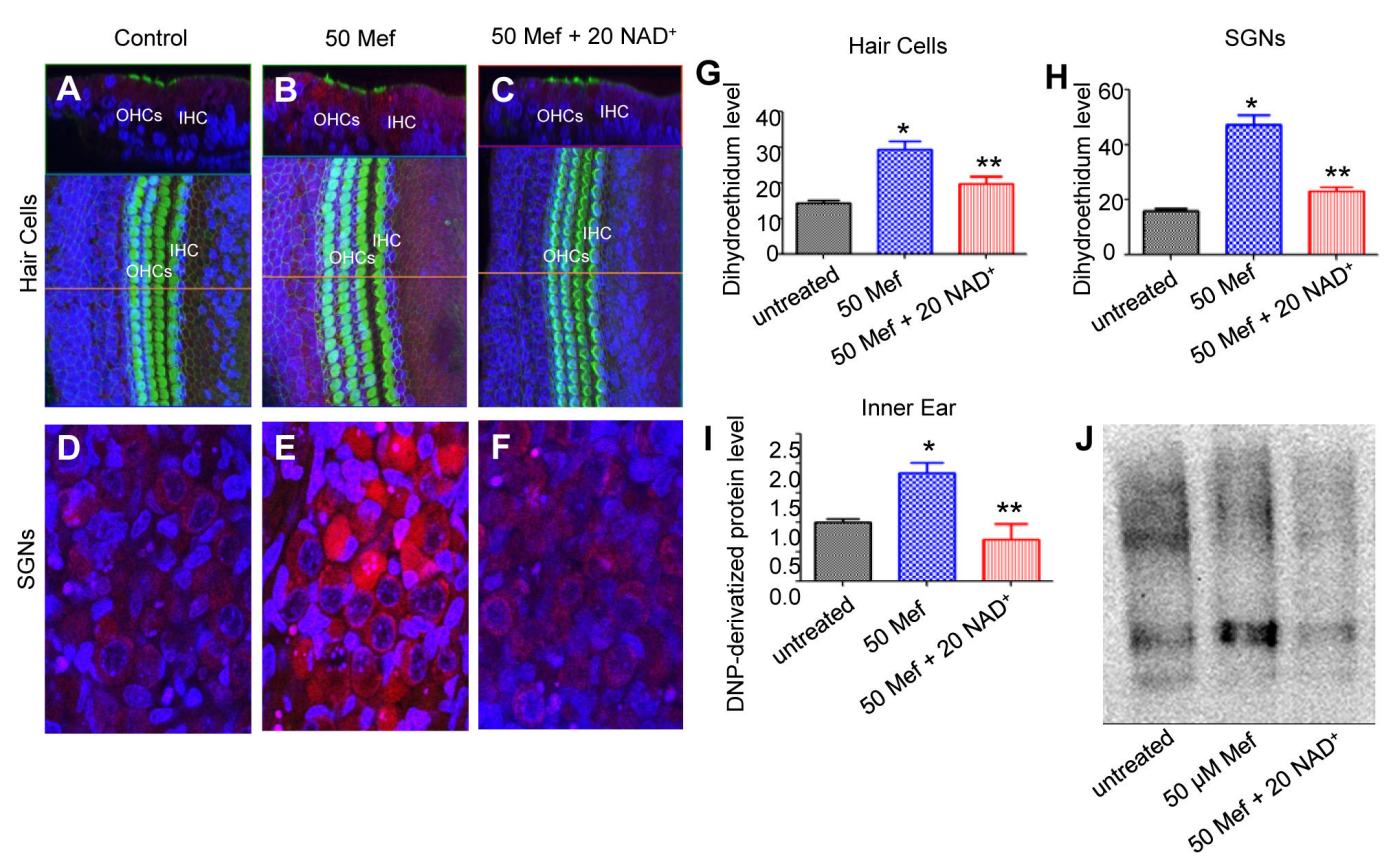
Mefloquine increases oxidative stress in the hair cells and spiral ganglion neurons, while NAD^+^ reduces mefloquine-induced oxidative stress in cultured cochleae. Dihydroethidium red fluorescence indicates superoxide production in hair cells in the vertical section of organ of Corti (upper panel) and surface preparation (lower panel) in cultured cochlea without mefloquine treatment (a), or treated with 50 µM mefloquine alone (b) or 50 µM mefloquine + 20 mM NAD^+^ (c). Spiral ganglion neurons in cultured cochlea without mefloquine treatment (d), or treated with 50 µM mefloquine alone (e) or 50 µM mefloquine + 20 mM NAD^+^ (f). Superoxide-positive cells were quantified in the hair cell regions (g) and spiral ganglion neuron regions (h). Oxidative protein damage (protein carbonyl) levels were measured by immunoblot in the homogenates of the cultured cochlea without mefloquine treatment, or treated with 50 µM mefloquine alone or 50 µM mefloquine + 20 mM NAD+ (i-j). *Significantly different from control (P < 0.05). **Significantly different from mefloquine treatment alone (P < 0.05). 50 Mef = 50 µM mefloquine, and 20 NAD+ = 20 mM NAD+.

A recent study has shown that mefloquine increases oxidative stress in primary rat cortical neurons [15]. Hence, we reasoned that mefloquine may cause cochlear degeneration through the induction of oxidative stress. To test this hypothesis, we measured the levels of superoxide, one of the major ROS, in the hair cells and spiral ganglion neurons of cultured cochlear organs treated with mefloquine alone or treated with both mefloquine and NAD^+^. We found that superoxide levels were increased in the hair cells and spiral ganglion neurons in 50 µM mefloquine-treated cochlear organs in comparison with untreated control (Figure 4a, b, d, e, g, and h). Strikingly, addition of 20 mM NAD^+^ significantly reduced superoxide levels in the hair cells and spiral ganglion neurons in the cochlear organs treated with 50 µM mefloquine (Figure 4c, f, g, and h). We then measured levels of protein carbonyl, a marker of protein oxidation, in cultured cochlea by immunoblotting. Treatment with 50 µM mefloquine for 12 hours significantly increased protein carbonyl levels compared to untreated controls (Figure 4i-j), while addition of 20 mM NAD^+^ significantly reduced levels of protein carbonyl in the hair cells and auditory nerve fibers from mefloquine-treated cochlear organs (Figure 4i-j). Together, these results suggest that mefloquine may cause degeneration of hair cells and auditory nerves through the induction of oxidative stress, and that NAD^+^ may protect sensory hair cells and auditory nerve fibers through reducing oxidative stress. 

**Figure 5:**
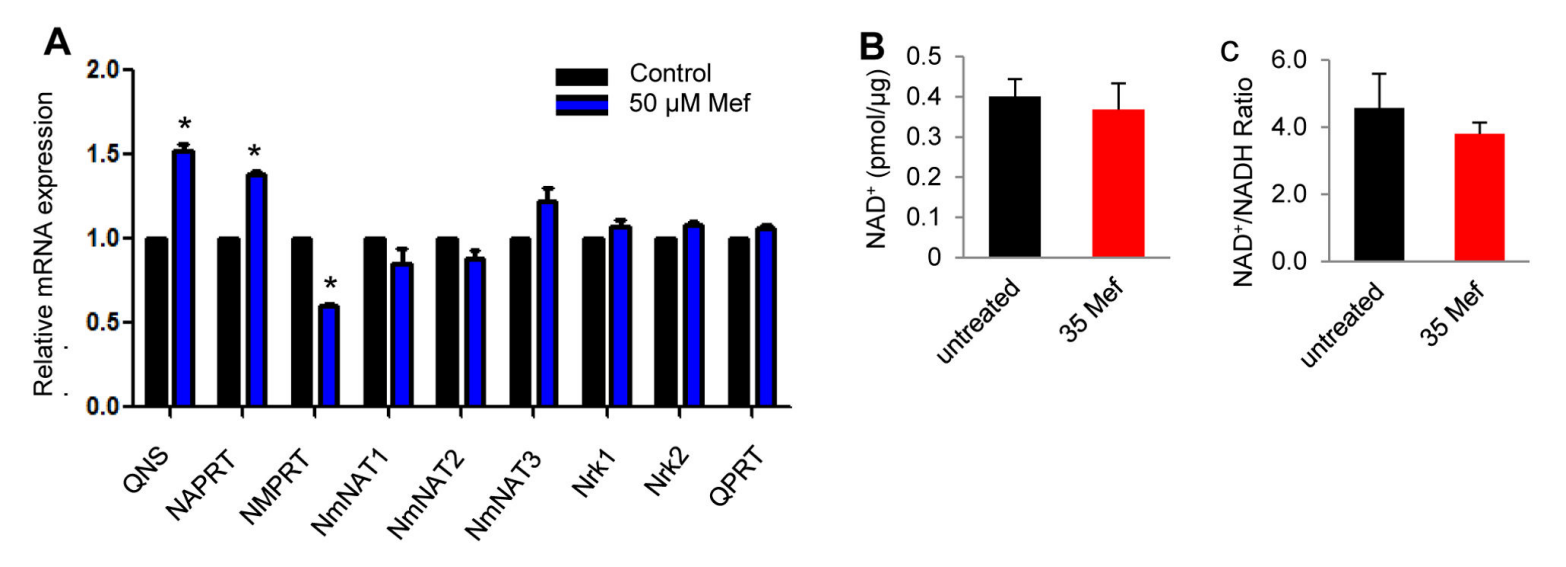
Mefloquine treatment does not affect NAD^+^ biosynthesis. mRNA expression levels of 9 genes involved in NAD^+^ biosynthesis were measured in cultured cochlea without mefloquine treatment, or treated with 50 µM mefloquine by quantitative RT-PCR. *Significantly different from control (a) (*P* < 0.05). NAD^+^ levels (b) and NAD^+^/NADH ratios (c) were measured in the homogenates of the cultured cochleae without mefloquine treatment (untreated), or treated with 35 µM mefloquine alone. 35 Mef = 35 µM mefloquine, and 20 NAD^+^ = 20 mM NAD^+^.

It is thought that NAD^+^ depletion causes cell death under cytotoxic stress [36]. Hence, we reasoned that mefloquine may induce degeneration of auditory nerve fibers and hair cells in cultured cochleae by suppressing or affecting NAD^+^ production. To test this hypothesis, we first investigated whether mefloquine treatment resulted in decreased gene expression of the enzymes involved in NAD^+^ biosynthesis by measuring the mRNA expression levels of the enzymes in cultured cochleae with or without mefloquine using quantitative RT-PCR. Off the 9 genes involved in NAD^+^ biosynthesis and tested in our assay, 2 genes (*NAPRT and QNS*) were up-regulated, while only 1 gene (*NMPRT*) was down-regulated by mefloquine treatment (Figure 5a). We then measured NAD^+^ levels and NAD^+^/NADP ratios in cultured cochleae with or without mefloquine. We found that mefloquine treatment did not decrease or affect NAD^+^ levels or NAD^+^/NADP ratios in the cochlear organ (Figure 5b-c). Therefore, these results suggest that although NAD^+^ may protect cochlear cells through reducing oxidative stress, mefloquine causes cochlear degeneration, not through affecting the NAD^+^ biosynthesis, but through the induction of oxidative stress. 

### 4: NAD^+^ reduces mefloquine-induced apoptosis in the auditory nerve fibers in cultured cochleae

**Figure 6:**
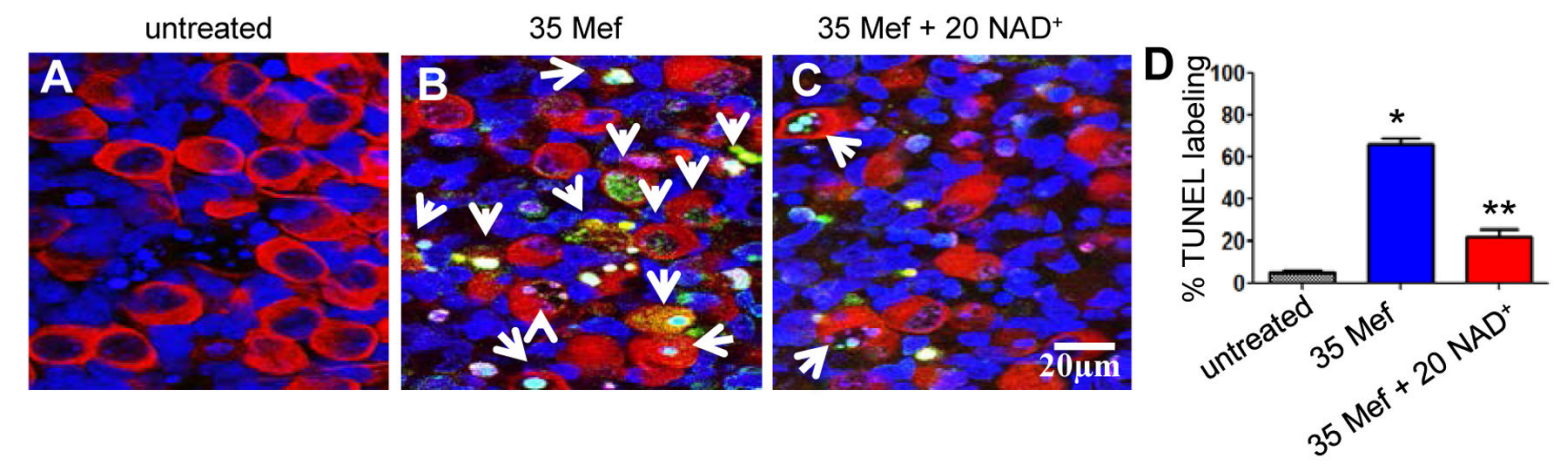
Mefloquine increases TUNEL-positive spiral ganglion neurons, while NAD^+^ prevents the increases. Green fluorescence indicates nuclear DNA fragmentation in the spiral ganglion neurons in cultured cochlea without mefloquine treatment (a), or treated with 35 µM mefloquine alone (b) or 35 µM mefloquine + 20 mM NAD^+^ (c). TUNEL-positive cells were quantified in the spiral ganglion neuron regions (d). *Significantly different from control (*P* < 0.05). **Significantly different from mefloquine treatment alone (*P* < 0.05). 35 Mef = 35 µM mefloquine, and 20 NAD^+^ = 20 mM NAD^+^. Arrows indicate TUNEL-positive cells.

**Figure 7:**
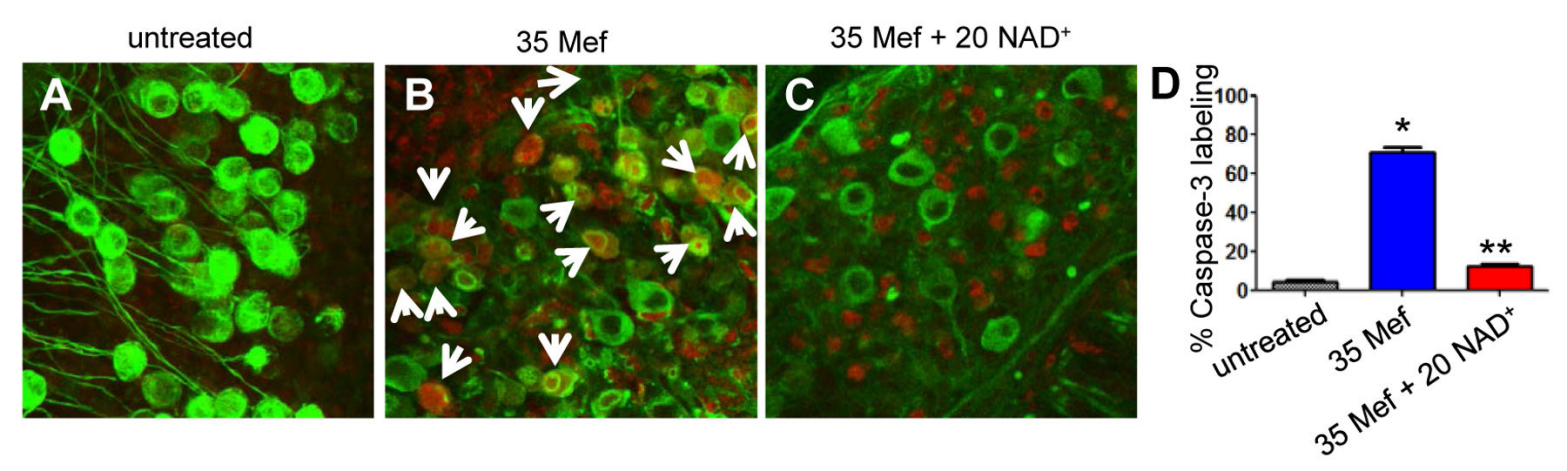
Mefloquine increases active caspase-3-positive spiral ganglion neurons, while NAD^+^ prevents the increases. Red fluorescence indicates active caspases-3 in the spiral ganglion neurons in cultured cochlea without mefloquine treatment (a), or treated with 35 µM mefloquine alone (b) or 35 µM mefloquine + 20 mM NAD^+^ (c). Active caspase-3-positive cells were quantified in the spiral ganglion neuron regions (d). *Significantly different from control (*P* < 0.05). **Significantly different from mefloquine treatment alone (*P* < 0.05). 35 Mef = 35 µM mefloquine, and 20 NAD^+^ = 20 mM NAD^+^. Arrows indicate caspases-3-positive cells.

A large body of evidence indicates that oxidative damage and associated apoptosis contribute to the development of Parkinson's disease, Alzheimer's disease, and other age-associated neurodegenerative diseases [37-39] . Hence, we reasoned that mefloquine-induced oxidative stress may trigger apoptosis, while NAD^+^ treatment may protect cochlear neurons through the reduction of apoptosis. To test this hypothesis, we measured the levels of two major apoptosis markers, nuclear DNA fragmentation and caspases-3, in the spiral ganglion neurons of cultured cochlear organs treated with mefloquine alone or mefloquine and NAD^+^. We found that mefloquine-treated cochleae displayed more TUNEL-positive spiral ganglion neurons compared to control cochlear organs (Figure 6a, b, d). In contrast, spiral ganglion neurons of cultured cochlear organs treated with both mefloquine and NAD^+^ displayed significantly fewer TUNEL-positive neurons compared to mefloquine-treated cochlear organs (Figure 6a, c, d). Consistent with the TUNEL staining results, we found that mefloquine-treated cochleae displayed more active caspases-3-positive spiral ganglion neurons compared to control cochlear organs, while spiral ganglion neurons of cultured cochlear organs treated with both mefloquine and NAD^+^ displayed significantly fewer active caspases-3-positive neurons compared to mefloquine-treated cochlear organs (Figure 7a-d). The NAD^+^ treatment appeared to protect the spiral ganglion cells, but not the non-neuronal cells. In this primary culture system, the spiral ganglion cells usually keep their normal morphology for several days, while the surrounding supporting cells such as Schwann cells or Glial cells may undergo dedifferentiation, and start to proliferate rapidly. This might explain why NAD^+^ protects the spiral ganglion cells, but the non-neuronal cells in cochlear cultures. Taken together, these results suggest that NAD^+^ treatment may protect cochlear neurons by blocking caspases-3-mediated apoptosis. 

## Discussion

### 1: Protection of cochlear auditory nerve and hair cells by NAD^+^

Previous studies have demonstrated that a decline in axonal NAD^+^ levels is a common feature of axonal degeneration triggered by various chemical insults [17,40,41]. Consistent with these reports, NAD^+^ is a substrate for Sir2 (silent information regulator 2), which regulates DNA damage repair, cell cycle, and life span extension in lower organisms [42-44]. In mammals, the NAD^+^-dependent deacetylase Sirt1 is thought to regulate cell survival through inhibiting p53-dependent apoptosis [19,45-47]. Moreover, NAD^+^ is a substrate for PARP [poly (ADP-ribose) polymerase], a protein involved in DNA repair and programmed cell death [48-50]. Consistent with these reports, decreased NAD^+^ levels are known to activate apoptotic signaling pathways and to cause cell death [17,40,51], while an increase in NAD^+^ levels protects axons from degeneration [17,18,40,51]. It is also well-established that mitochondrial-mediated apoptosis can be induced by oxidative stress, and that this intrinsic apoptosis pathway involves the degradation of cellular components by caspases-3 [52-54]. In the current study, we have demonstrated that mefloquine treatment increases nuclear DNA fragmentation and active caspases-3 levels, key features of apoptosis, in the spiral ganglion neurons of cultured cochlear organs, while treatment with NAD^+^ prevents the increases. Therefore, we speculate that NAD^+^ treatment may activate Sirt1, which in turn reduce auditory nerve and/or hair cell degeneration through the inhibition of p53-mediated and/or mitochondrial caspases-3-mediated apoptosis in mefloquine-treated cochlear organs. 

Another potential explanation for the protection of cochlear cells by NAD^+^ comes from the observation that damaged cells display a reduction in ATP and NAD^+^ levels, implying that NAD depletion may impair energy production [51], while another study has shown that NAD^+^ treatment prevents the decline of ATP levels [51]. Hence, it is possible that NAD^+^ treatment may reduce auditory nerve and/or hair cell degeneration by preventing energy depletion in the cochlea which requires high levels of ATP. 

### 2: Mefloquine causes cochlear degeneration through the induction of oxidative stress

**Figure 8:**
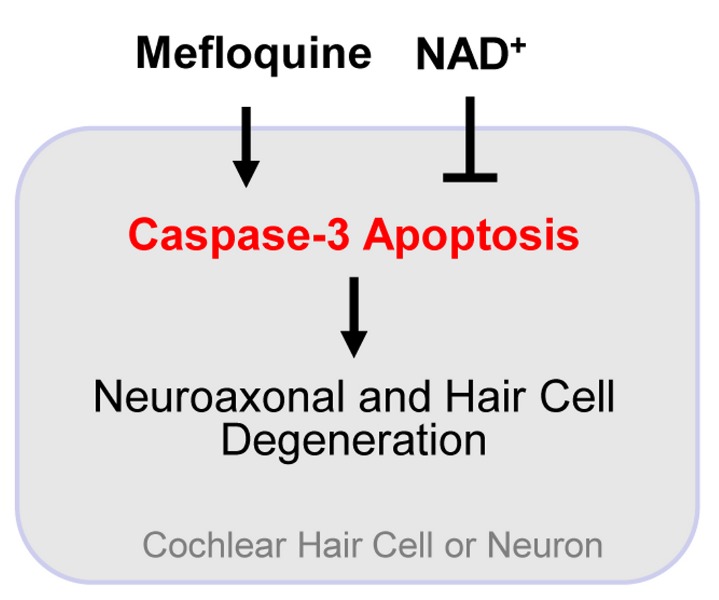
A model for the NAD^+^-mediated prevention of mefloquine-induced neuroaxonal and hair cell degeneration. In this model, caspases-3-mediated apoptosis plays an important mechanism linking mefloquine, cochlear neuroaxonal and hair cell degeneration, and NAD^+^.

It is thought that degeneration of auditory nerve axons occurs early and proceeds quickly after mefloquine exposure [10-13], and the first cytotoxic effect of mefloquine is thought to result from free heme accumulation that is responsible for its antimalarial effects [55]. Consistent with this report, it is well-established that free heme is a cytotoxic iron compound that causes the formation of ROS [56]. Hence, the cytotoxic mechanism by which mefloquine causes neurodegeneration is likely through generation of free heme, which then acts as a catalyst to form ROS [15,57]. In the present study, we have demonstrated that mefloquine treatment causes degeneration of cochlear hair cells and auditory nerves in a dose-dependent manner, and that it increases superoxide and protein carbonyl levels in the hair cells and spiral ganglion neurons in the cochleae. Therefore, we speculate that mefloquine-induced degeneration of the cochlear auditory nervous system is triggered by an increase in oxidative stress. In summary, our findings provide evidence that caspases-3-mediated apoptosis may be an important mechanism linking mefloquine, cochlear neuroaxonal and hair cell degeneration, and NAD^+^, and that NAD^+^ is a useful drug candidate in reducing or slowing the degeneration of cochlear axons and sensory hair cells caused by mefloquine treatment (Figure 8).
